# Contemporary Theory and Research

**Published:** 1893-06-17

**Authors:** 


### Contemporary Theory and Research.

### HABITUAL DRUNKARDS.

A contemporary strongly advocates the compulsory restraint of habitual drunkards "at once for the benefit of the inebriate and in order to mitigate the intolerable misery from which his family in countless instances suffers." The following is a copy of the memorial signed by upwards of eighty influential physicians and surgeons and presented to the Departmental Committee appointed by the Home Office to consider the question of compulsory legislation. "We, the undersigned members of the medical profession, having had experience of the great difficulty which now exists in dealing with inebriates, respectfully urge upon the Committee the pressing need of further legislation concerning them. At present provision is made to a very limited extent by the Inebriates' Acts, the essential feature of which is that a person may place himself under restraint for a period not exceeding a twelvemonth. This has been found to be of limited value; the proportion of persons, especially ladies, who will go before magistrates and voluntarily place themselves under restraint is small, and we think that unless the clause is repealed which requires appearance before two justices, the Acts will remain of little value. We earnestly desire the compulsory restraint, with all proper safeguards, of those men and women who cannot control themselves in this respect. We are of opinion that much good may be done to inebriates if compulsory detention can be enforced for a sufficient time, and if upon release and subsequent breakdown, they can again be placed under control without delay or difficulty." The whole question is one bristling with difficulties. Compulsory legislation of the kind here advocated is open to the gravest objections, not only as tending to bear almost exclusively on the poorer classes who are unable to hide their excesses, but as opening a possibility of police inquisition of an intolerable description.

### POISONED ARROWS.

Dr. Le Dantec in a work on the telluric origin of the poison of arrows gives an account of recent researches into the history of this question, the origin of the poison, and the treatment of the wounds. He has subjected the arrows to careful examination and experiment in his laboratory. The war arrows of the natives of the New Hebrides are composed of a reed containing a centre of hard wood with a fragment of human bone, carefully scraped so as to form a delicate point. This point is covered with a black plaster, which constitutes the poison. From the experiments which have been made it seems that these arrows are poisoned with the earth of the marshes. This earth contains two pathogenic microbes—the septic vibrion and the bacillus of tetanus. Dr. Le Dantec observes that these experiences are an argument against the equine origin of tetanus, since there have never been any horses in the New Hebrides.

